# CALM-CXR: a calibration-aware lung-masked workflow for hierarchical chest X-ray classification

**DOI:** 10.1016/j.mex.2026.104081

**Published:** 2026-07-31

**Authors:** Suresh Kumar Samarla, D.N.S.B. Kavitha, Solleti Phanikumar, Boddu L.V. Siva Rama Krishna, D.S.S. Lakshmi Kumari P

**Affiliations:** a Department of Computer Science and Engineering, Sagi Rama Krishnam Raju Engineering College, Bhimavaram, Andhra Pradesh, India; b Computer Science and Engineering, Koneru Lakshmaiah Education Foundation, Vaddeswaram, Andhra Pradesh, India; c Department of Computer Science and Engineering, SRM University-AP, Amaravati, Andhra Pradesh, India; d Department of Information Technology, Sagi Rama Krishnam Raju Engineering College, Bhimavaram, Andhra Pradesh, India

**Keywords:** Hierarchical deep learning, Lung masking, Uncertainty calibration, Temperature scaling, Medical image analysis

## Abstract

Chest X-ray classification studies often report endpoint performance without isolating how anatomical guidance, staged decisions, probability calibration, and error propagation influence the final prediction. CALM-CXR is a reproducible protocol that operationally couples lung-mask-conditioned regional expert modeling with separately trained normal-abnormal and bacterial-viral stages, stage-specific temperature scaling, calibration-partition threshold selection, and coherent three-class probability composition. Its methodological advance is the controlled, auditable coupling of these established components: each stage retains its own probability and decision parameters, final class probabilities remain mathematically coherent, and errors can be attributed to screening or subtype classification. The protocol was evaluated on 4,840 images using filename-derived group-disjoint train, validation, calibration, and test partitions. Across seeds 42, 52, and 62, the full protocol achieved accuracy 0.7920 (SD 0.0248), macro-F1 0.7999 (SD 0.0165), macro one-vs-rest AUROC 0.9063 (SD 0.0088), and macro PR-AUC 0.8616 (SD 0.0068). Removing lung-mask conditioning produced the largest mean decrease in accuracy (0.0234) and macro-F1 (0.0206) among the component ablations. A matched flat ConvNeXt-Tiny classifier achieved accuracy 0.7955 (SD 0.0135) and macro-F1 0.7982 (SD 0.0128), and paired comparisons did not establish consistent overall superiority of the hierarchical organization. CALM-CXR should therefore be interpreted as an auditable evaluation protocol, not as a new individual primitive or a clinically validated diagnostic system, independent external validation remains necessary.

➢ Defines an auditable two-stage evaluation protocol that separates normal-abnormal screening from bacterial-viral subtyping and retains stage-specific probabilities and errors.

➢ Tests lung-mask conditioning, learned regional routing, anatomy and routing-entropy penalties, temperature scaling, calibration-only thresholds, and hierarchical probability composition through controlled ablations.

➢ Includes matched flat three-class baselines, paired statistical comparisons, calibration metrics, bootstrap uncertainty, and filename-derived group-overlap auditing.

## Graphical abstract

**Figure:**
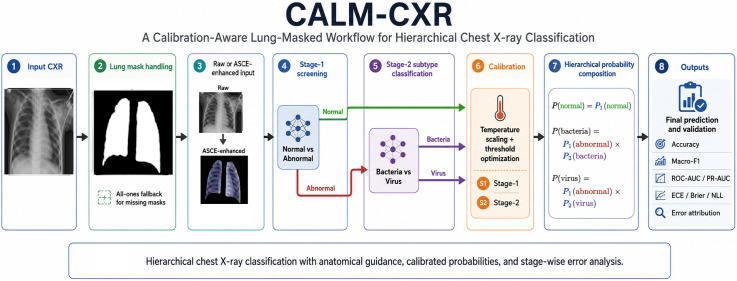

## Specifications table

**Table:** 

**Subject area**	Computer Science
**More specific subject area**	*Reproducible deep learning workflow for calibrated and hierarchical chest X-ray classification*
**Name of your protocol**	*Calibration-Aware Lung-Masked Chest X-ray classification workflow*
**Reagents/tools**	*Python 3, PyTorch, torchvision, scikit-learn, NumPy, pandas, matplotlib, Google Colab or GPU-enabled Python environment*
**Experimental design**	*Chest X-ray images are organized into bacteria, normal, and virus categories.* *The protocol uses a two-stage design: Stage-1 performs normal versus abnormal screening, and Stage-2 performs bacterial versus viral classification among abnormal samples. Final class probabilities are generated using hierarchical probability composition.*
**Trial registration**	*Not applicable*
**Ethics**	*The protocol is intended for anonymized chest X-ray image datasets. No direct human subject interaction is involved. If a private clinical dataset is used, institutional ethics approval and data-use permission must be obtained before applying the protocol.*
**Value of the Protocol**	1. *Provides a stage-auditable framework: Stage 1 screens normal versus abnormal and Stage 2 subtypes abnormal images, allowing screening and subtype failures to be examined separately.* 2. *Operationally couples lung-mask-conditioned regional experts, anatomy and routing-entropy regularization, stage-specific calibration, calibration-only thresholds, and coherent final probability composition. These are established methods, the contribution is their controlled coupling and testable computational roles.* 3. *Supports direct component ablations and matched flat three-class baselines under the same filename-derived group-disjoint split, with discrimination, calibration, bootstrap uncertainty, paired prediction-level comparisons, and explicit avoidance of unsupported patient-level claims.*

## Background

Thoracic disease screening and follow-up is one of the most commonly employed imaging modalities that uses chest radiography. Automated interpretation of chest X-rays, therefore, is an important field of research in medical image analysis. Deep-learning systems have demonstrated high performance for chest radiograph diagnosis such as multi-label thoracic abnormality detection and comparison to practicing radiologists [1]. These systems must also be validated carefully, however, due to the subtle nature of the radiographic findings, and overlap in the findings from different datasets, image characteristics can influence the results. Rajpurkar et al. [1] reported a retrospective comparison of the CheXNeXt algorithm to practicing radiologists, demonstrating the potential and caution for interpreting deep-learning algorithms for chest radiograph reading.

Recently, the literature on pneumonia detection has shifted from a binary classification to more complex multi-class classification, with some methodological issues yet to be addressed. These include dataset bias, class imbalance, limited code availability, incompleteness of model comparison, difficulty of explanation, and generalization across datasets. These concerns are crucial in protocol design, as the protocol should not only make a final prediction but also make the evaluation procedure repeatable and verifiable. In particular, Siddiqi and Javaid's 2024 survey highlights [2] the ongoing demand for systematic comparisons of models, explainability, code availability and addressing class-imbalances in the context of pneumonia detection in CXR.

Most of the X-ray classification workflows are flat classifiers where all categories are classified in a single classification step. However, the interpretation of radiographs tends to be staged. The reader is asked to make the initial decision of whether the radiograph is normal or abnormal and then to think in terms of more specific disease types. A staged reasoning is helpful in computational workflows because it enables the model to be examined individually at each decision level. The classification task is thus divided into Stage-1 screening of normal versus abnormal, and Stage-2 bacterial-versus-viral subtyping, in the proposed protocol. This design is not assuming that bacterial and viral pneumonia can always be distinguished from the radiograph but offers a clear framework for assessing the extent to which the workflow is successful and the extent to which it fails.

Secondly, there is the anatomical validity issue. Chest radiography consists of lung fields, bones, acquisition markers, borders and non-lung background regions. Anatomical guidance is needed to ensure that a model does not learn correlations from regions that are not clinically meaningful. Therefore, the segmentation of the lungs can be helpful as a pre-processing or guidance step in lung X-ray workflows. The importance of lung region extraction in CXR analysis is corroborated by the recent research in this field, like CXR-Seg [3], which has focused on dedicated deep-learning models for lung segmentation from CXR images. The handling of anatomical regions is made explicit and reproducible in the present protocol, by making use of lung-mask handling and optionally using ASCE enhancement.

Another point of concern is annotation and localization. A limited ability to assess if the model was using relevant image regions is obtained in some chest X-ray datasets, where these are only provided at the image level. The VinDr-CXR [4] dataset article mentions that there are numerous datasets of CXR with finding labels in which their location on the image is not specified, and introduces a large radiologist-annotated chest X-ray dataset for detection and localization research. This reinforces the requirement for workflows that account for anatomical guidance, region handling, and how errors are handled, in addition to simply reporting accuracy.

A fourth issue is interpretability and reliability. Studies on explainable CXR have examined region-based or texture-based analysis to gain insight into the regions that make decisions in the classification task. In COVID-19 pneumonia classification using chest radiographs, Moura et al. [5] demonstrated the importance of exploring discriminative evidence of the lung regions using texture-based features and explainable machine-learning analysis. Reliability in the sense of probability is also a factor, besides interpretability. Even if a model can classify a large number of images correctly, it can still give poorly calibrated probabilities. Various warnings like Brier score [6], Negative Log-Likelihood and Expected Calibration Error provide an evaluation for meaningful probability outputs as confidence estimates.

The proposed CALM-CXR protocol overcomes these issues by addressing each of them in the following sequence: mask-guided input handling, optional ASCE enhancement [7], two-stage hierarchical classification, probability calibration by temperature, error threshold optimization, hierarchical probability composition and stage-wise error attribution. none is claimed as a new standalone method. The methodological advance of CALM-CXR is their controlled, auditable coupling. Each element has a defined computational role and a corresponding ablation, the two stages retain separate calibration parameters, hierarchical composition propagates the Stage 1 abnormal probability into subtype probabilities, and errors can be attributed to screening or subtyping. The workflow aims to enable researchers to replicate, assess, and compare anatomy-aware and calibration-aware pipelines for classification of chest X-ray images based on a well-defined experimental protocol.

## Description of protocol

CALM-CXR is a reproducible evaluation protocol for the three dataset labels bacteria, normal, and virus. It separates model fitting, model selection, probability calibration, threshold selection, and final test evaluation. The hierarchical organization exposes the normal-abnormal screening stage and the bacteria-virus subtype stage separately, it is not assumed to be intrinsically more accurate than flat three-class classification.

### Required inputs and primary-analysis manifest

The implementation requires three image folders named bacteria, normal, and virus, a lung-mask directory indexed by image stem, and a manifest containing the image path, class label, and split-group identifier. The final reviewer analysis used 4,840 stored ASCE-processed images: 2,587 bacteria, 857 normal, and 1,396 virus images. The manifest contained 2,481 distinct split_group_id values.

These split identifiers were derived from documented filename patterns and were not authenticated against clinical patient records. Accordingly, the revised manuscript reports a filename-derived group-disjoint split rather than a verified patient-level split. A further 176 images without an acceptable filename-derived grouping basis were excluded from the primary analysis, their metadata and near-duplicate audit remain in the supplementary package.

### Software, configuration, and execution controls

The final run used Python 3.12.13, PyTorch 2.11.0 with CUDA 12.8, torchvision 0.26.0, NumPy 2.0.2, pandas 2.2.2, and a CUDA-enabled GPU. The complete package records the platform, cuDNN version, dependency environment, configuration, code hash, dataset hash, and split hash. The archived reviewer source and core module reproduce the 24-character code hash stored in the final environment manifest.

input_size = 224; backbone = convnext_tiny; grid_size = 7

num_experts = 3; expert_dim = 256; router_hidden = 256; mc_samples = 6

batch_size = 16; epochs_stage1 = 12; epochs_stage2 = 12; epochs_flat = 20

split_seed = 42; model_seeds = (42, 52, 62); bootstrap_resamples = 500

One signed master split was shared by every model seed and experiment. The runner sets Python, NumPy, PyTorch, CUDA, DataLoader, and sampler random states; writes epoch-level model, optimizer, scaler, and random-state checkpoints; caches logits; validates experiment and evaluation signatures; and reuses only matching completed artifacts. The final notebook also records the two runtime corrections used during execution: uniform-routing ablations disable uncertainty-dependent routing temperature, and restored random-state tensors are converted to CPU byte tensors before generator restoration.

### Data partition

Split groups were assigned with a fixed split seed of 42 using the nominal proportions 70% training, 10% validation, 10% calibration, and 10% testing. Group-level stratification used the majority class of each group, and every image from a group remained in one partition. Because groups are indivisible, the realized image counts differ slightly from the nominal proportions.

The realized partitions were: training, 3,434 images from 1,736 groups; validation, 435 images from 248 groups; calibration, 487 images from 248 groups; and testing, 484 images from 249 groups. The stored audit found no split_group_id overlap among any pair of partitions. The training partition updated model parameters, validation macro-F1 selected checkpoints, the calibration partition fitted temperatures and thresholds, and the test partition was not used for fitting or selection.

### Hierarchical task construction

Stage 1 maps normal images to 0 and bacteria or virus images to the abnormal label 1. Stage 2 is trained only on abnormal images, mapping bacteria to 0 and virus to 1. The final output order is bacteria, normal, virus. These names are dataset categories and do not establish microbiological etiology from a radiograph.

For probability completion on normal test images, for which Stage 2 has no defined sample, the implementation uses a neutral Stage-2 vector [0.5, 0.5]. Its contribution is multiplied by the Stage-1 abnormal probability and therefore does not replace the Stage-1 normal probability.

The three-class label y∈{bacteria,normal,virus}is converted into two stage-specific binary labels:

(1) $$y^{\left(1\right)}=\left\{ \begin{array}{cc} 0, & y=\text{normal},\\ 1, & y\in \left\{\text{bacteria},\text{virus}\right\}, \end{array}\right.y^{\left(2\right)}=\left\{ \begin{array}{cc} 0, & y=\text{bacteria},\\ 1, & y=\text{virus}. \end{array}\right.$$

Here, y is the original three-class dataset label. The Stage-1 label $y^{\left(1\right)}$represents normal (0) versus abnormal (1) screening. The Stage-2 label $y^{\left(2\right)}$represents bacterial (0) versus viral (1) classification and is defined only for abnormal images

### Image and lung-mask preprocessing

Images are converted to RGB, resized to 224 × 224 pixels, transformed to tensors, and normalized with the ImageNet channel statistics used by the pretrained backbone. Masks are matched by filename stem, resized with nearest-neighbor interpolation, converted to tensors, and binarized at 0.05. The primary analysis used the provided-mask mode and stored ASCE-processed images with enhance_on_fly set to false.

If a mask is absent, the loader returns an all-ones mask rather than a zero mask, preventing mask availability from becoming a label cue. The controlled no-lung-mask ablation applies an all-ones mask to every sample, thereby removing spatial lung conditioning while preserving the remaining data and training pipeline.

### Mask-conditioned regional expert computation

A pretrained ConvNeXt-Tiny backbone produces a feature map. The resized lung mask is multiplied with this map, after which adaptive average pooling forms a 7 × 7 grid of 49 regional descriptors. Each descriptor is projected to 256 dimensions. A second pooled mask records lung occupancy for every grid cell, and an occupancy-weighted mean supplies the global context vector.

Let the backbone feature map for image $x_{i}$be

(2) $$F_{i}=f_{\theta }\left(x_{i}\right),F_{i}\in R^{C^{\prime }\times H^{\prime }\times W^{\prime }}$$

where $f_{\theta }$denotes the ConvNeXt-Tiny feature extractor with learnable parameters θ. The lung mask is resized to the feature-map resolution,

(3) $$M_{i}^{{}^{\prime }}=\text{Resize}\left(M_{i},H^{\prime },W^{\prime }\right),$$

and mask conditioning is applied before regional pooling:

(4) $$F_{m,i}=F_{i}⊙M_{i}^{{}^{\prime }}.$$

Here, $x_{i}$is the i-th chest X-ray, $M_{i}$is its binary or soft lung mask, and $F_{i}$is the corresponding backbone feature map. The dimensions $C^{\prime }$, $H^{\prime }$, and $W^{\prime }$denote the number of feature channels, height, and width, respectively. The symbol ⊙denotes element-wise multiplication, with $M_{i}^{{}^{\prime }}$broadcast across the feature channels. The resulting $F_{m,i}$is the lung-mask-conditioned feature map. daptive average pooling converts the mask-conditioned feature map into a g×ggrid. For the implemented g=7, this produces $N=g^{2}=49$regional descriptors. After projection to the expert dimension, the regional descriptor and lung occupancy for cell nare

(5) $$g_{in}=\phi \;\left({\left[\text{AAP}{}_{g\times g}\left(F_{m,i}\right)\right]}_{n}\right),m_{in}={\left[\text{AAP}{}_{g\times g}\left(M_{i}^{{}^{\prime }}\right)\right]}_{n},$$

where ϕ(·)is the learned projection to 256 dimensions. An occupancy-weighted global context vector is then computed as

(6) $$c_{i}=\frac{\sum_{n=1}^{N}m_{in}g_{in}}{\sum_{n=1}^{N}m_{in}+\varepsilon }.$$

Here, iindexes images, n∈{1,…,N}indexes regional grid cells, $g_{in}\in R^{256}$is the projected descriptor of region n, and $m_{in}\in \left[0,1\right]$is the corresponding lung occupancy. The operator $\text{AAP}{}_{g\times g}$denotes adaptive average pooling, $c_{i}$is the global lung-supported context vector, and εis a small constant used for numerical stability.

The learned router is a dropout-enabled multilayer perceptron. During training, six routing passes produce mean assignments over three experts, assignment variance, routing entropy, and a regional epistemic score. This score maps routing temperature between 0.70 and 1.50 before lung-occupancy-weighted expert pooling. Calibration and test logits are collected with one deterministic routing pass; the stored test results therefore do not represent Monte Carlo uncertainty averaging. Expert representations are exchanged through four-head cross-expert attention and fused with the global context to produce the stage logits.

During training, the router is evaluated using S=6stochastic dropout passes. For image i, regional cell n, expert k, and stochastic pass s, the mean expert-assignment probability is

(7) $${\overset{ˉ}{\pi }}_{ink}=\frac{1}{S}\sum_{s=1}^{S}{\left[\text{softmax}\left(r_{ins}\right)\right]}_{k},$$

and the regional epistemic routing score is

(8) $$u_{in}=\sum_{k=1}^{K}\text{Var}{}_{s}\left({\left[\text{softmax}\left(r_{ins}\right)\right]}_{k}\right).$$

Here, $r_{ins}\in R^{K}$is the router-logit vector obtained during stochastic pass s, K=3is the number of expert branches, and ${\overset{ˉ}{\pi }}_{ink}$is the mean probability of assigning regional cell nto expert k. The quantity $u_{in}$is the sum of the pass-wise assignment variances across the experts. Calibration and test logits are collected using one deterministic routing pass (S=1); therefore, the stored test results do not represent Monte Carlo uncertainty averaging.

The regional routing temperature is obtained by normalizing $u_{in}$within each image and mapping it to the configured interval:

(9) $$T_{in}=t_{\text{min}}+\left(t_{\text{max}}-t_{\text{min}}\right)\frac{u_{in}-\underset{n^{\prime }}{\text{min}}u_{in^{\prime }}}{\underset{n^{\prime }}{\text{max}}u_{in^{\prime }}-\underset{n^{\prime }}{\text{min}}u_{in^{\prime }}+\varepsilon },$$

where $t_{\text{min}}=0.70$and $t_{\text{max}}=1.50$. The adjusted routing weights are

(10) $$q_{ink}=\frac{\text{exp}\;\left[\text{log}\left({\overset{ˉ}{\pi }}_{ink}+\varepsilon \right)/T_{in}\right]}{\sum_{j=1}^{K}\text{exp}\;\left[\text{log}\left({\overset{ˉ}{\pi }}_{inj}+\varepsilon \right)/T_{in}\right]}.$$

The lung-occupancy-weighted input to expert kand its resulting representation are

(11) $$v_{ik}=\frac{\sum_{n=1}^{N}q_{ink}m_{in}g_{in}}{\sum_{n=1}^{N}q_{ink}m_{in}+\varepsilon },e_{ik}=E_{k}\left(v_{ik}\right).$$

Here, $T_{in}$is the uncertainty-dependent routing temperature for cell n, while $t_{\text{min}}$and $t_{\text{max}}$are its lower and upper bounds. The quantity $q_{ink}$is the temperature-adjusted routing weight assigned to expert k. The vector $v_{ik}$is the lung-occupancy-weighted regional summary provided to expert $E_{k}$, and $e_{ik}$is the resulting expert representation. The index jis used in the normalization across all Kexperts.

After this definition, retain the existing explanation that the expert representations undergo four-head cross-expert attention and are fused with the global context vector.

In the corrected no-regional-routing experiment, every grid cell is assigned uniformly to the three experts and the uncertainty-dependent routing temperature is disabled. This isolates learned regional allocation without introducing undefined uncertainty normalization.

### Loss and stage-wise training

Each binary stage is optimized independently using class-weighted cross-entropy with label smoothing 0.05. The complete training objective adds an off-lung anatomy term, an in-lung routing-entropy term, and the mean conflict output:

The entropy of the mean routing distribution at regional cell nis

(12) $$H\left({\overset{ˉ}{\pi }}_{in}\right)=-\sum_{k=1}^{K}{\overset{ˉ}{\pi }}_{ink}\text{log}\left({\overset{ˉ}{\pi }}_{ink}+\varepsilon \right).$$

The routing-entropy penalty is evaluated primarily over lung-supported grid cells:

(13) $$L_{\text{entropy}}=\frac{\sum_{i}\sum_{n=1}^{N}m_{in}H\left({\overset{ˉ}{\pi }}_{in}\right)}{\sum_{i}\sum_{n=1}^{N}m_{in}+\varepsilon }.$$

The anatomy penalty measures confident expert assignment outside the lung-supported regions:

(14) $$L_{\text{anatomy}}=\frac{\sum_{i}\sum_{n=1}^{N}\left(1-m_{in}\right)\underset{k}{\text{max}}{\overset{ˉ}{\pi }}_{ink}}{\sum_{i}\sum_{n=1}^{N}\left(1-m_{in}\right)+\varepsilon }.$$

Here, ${\overset{ˉ}{\pi }}_{in}=\left[{\overset{ˉ}{\pi }}_{in1},\ldots ,{\overset{ˉ}{\pi }}_{inK}\right]$is the mean routing distribution for regional cell n. Its entropy $H({\overset{ˉ}{\pi }}_{in})$is high when routing is diffuse across experts and low when routing is concentrated. The quantity $m_{in}$weights the entropy term toward lung-supported cells, whereas $1-m_{in}$weights the anatomy term toward off-lung cells. The maximum routing probability measures the confidence of the strongest expert assignment.

The anatomy term penalizes confident expert assignment in grid cells with low lung occupancy. Minimizing the entropy term discourages diffuse in-lung routing. The conflict term is an auxiliary scalar regularizer. The anatomy and entropy coefficients are set to zero in their respective reviewer ablations; the conflict term was not separately ablated.

The complete objective used to train each binary stage is

(15) $$L_{\text{total}}=L_{\text{CE}}+0.05\;L_{\text{anatomy}}+0.01\;L_{\text{entropy}}+0.01\;L_{\text{conflict}}.$$

Here, $L_{\text{CE}}$is the class-weighted cross-entropy loss computed with label smoothing of 0.05. The terms $L_{\text{anatomy}}$and $L_{\text{entropy}}$are defined in Eqs. (13) and (14). The term $L_{\text{conflict}}$is the mean auxiliary conflict output. In the corresponding ablation experiments, the coefficient of either the anatomy or entropy term was set to zero while the remaining training configuration was retained. The conflict term was not evaluated through a separate ablation.

Training uses AdamW with learning rates 1 × 10^-5 for the backbone and 2 × 10^-4 for the remaining parameters, weight decay 1 × 10^-4, mixed precision, and a weighted replacement sampler. The backbone is frozen for the first three epochs and then unfrozen. For each stage and seed, the checkpoint with the highest validation macro-F1 is retained.

### Calibration, thresholds, and probability composition

After checkpoint selection, a separate scalar temperature is fitted to the calibration logits of each stage by minimizing cross-entropy. Stage-specific decision thresholds are then searched from 0.05 to 0.95 in increments of 0.005 and selected by calibration macro-F1. The no-temperature experiment fixes both temperatures at 1, and the fixed-threshold experiment uses 0.5 at both stages.

For stage s∈{1,2}, the calibrated probability assigned to class cis

(16) $${\tilde{p}}_{ic}^{\left(s\right)}\left(T_{s}\right)=\frac{\text{exp}\;\left(z_{ic}^{\left(s\right)}/T_{s}\right)}{\sum_{j=1}^{2}\text{exp}\left(z_{ij}^{\left(s\right)}/T_{s}\right)}.$$

The stage-specific temperature is fitted exclusively on the calibration partition C:

(17) $$T_{s}^{*}=\text{arg}\text{min}0.05\leq T\leq 20\left[-\sum_{i\in C}\text{log}{\tilde{p}}_{i,y_{i}}^{\left(s\right)}\left(T\right)\right].$$

Here, $z_{ic}^{\left(s\right)}$is the uncalibrated logit for sample i, class c, and stage s. The quantity $T_{s}>0$is the scalar temperature for stage s, and ${\tilde{p}}_{ic}^{\left(s\right)}$is the corresponding calibrated probability. The set Ccontains only calibration-partition samples, and $y_{i}$is the correct stage-specific label. The fitted temperature is bounded between 0.05 and 20, as implemented in the released code. Setting $T_{s}=1$produces the no-temperature-scaling ablation.

Let $P_{1}\left(\text{normal}|x\right)$and $P_{1}\left(\text{abnormal}|x\right)$denote the calibrated Stage-1 probabilities. Let $P_{2}\left(\text{bacteria}|x,\text{abnormal}\right)$and $P_{2}\left(\text{virus}|x,\text{abnormal}\right)$denote the calibrated Stage-2 probabilities. The final three-class probabilities are

(20) $$\begin{array}{cc} P\left(\text{normal}|x\right) & =P_{1}\left(\text{normal}|x\right),\\ P\left(\text{bacteria}|x\right) & =P_{1}\left(\text{abnormal}|x\right)P_{2}\left(\text{bacteria}|x,\text{abnormal}\right),\\ P\left(\text{virus}|x\right) & =P_{1}\left(\text{abnormal}|x\right)P_{2}\left(\text{virus}|x,\text{abnormal}\right). \end{array}$$

Here, xis the input image. The Stage-1 abnormal probability controls the total probability assigned to the two pneumonia categories. The Stage-2 conditional probabilities divide this abnormal probability between the bacteria and virus dataset labels. Because the two Stage-1 probabilities sum to one and the two Stage-2 probabilities sum to one, the composed three-class vector is also normalized:

(21) P(normal∣x)+P(bacteria∣x)+P(virus∣x)=1

This normalization identity provides the mathematical justification for describing the composition as probabilistically coherent.

Although the final probability vector is obtained through soft hierarchical composition, the stored class label follows the two stage-specific thresholds:

(22) $$\hat{y}=\left\{ \begin{array}{cc} \text{normal}, & P_{1}\left(\text{abnormal}|x\right)<\tau_{1}^{*},\\ \text{bacteria}, & P_{1}\left(\text{abnormal}|x\right)\geq \tau_{1}^{*}\;\text{and}\;P_{2}\left(\text{virus}|x,\text{abnormal}\right)<\tau_{2}^{*},\\ \text{virus}, & P_{1}\left(\text{abnormal}|x\right)\geq \tau_{1}^{*}\;\text{and}\;P_{2}\left(\text{virus}|x,\text{abnormal}\right)\geq \tau_{2}^{*}. \end{array}\right.$$

Here, $\hat{y}$is the final predicted dataset label, while $\tau_{1}^{*}$and $\tau_{2}^{*}$are the Stage-1 and Stage-2 thresholds selected exclusively from the calibration partition. The hard-cascade probability ablation retains this decision rule but replaces the soft composed probability vector. Consequently, that ablation leaves thresholded labels unchanged by design while affecting probability-ranking and calibration measures.

### Component ablations and matched flat-classifier baselines

Each of five structural conditions was trained independently for seeds 42, 52, and 62 on the same master split: full protocol, no lung mask, uniform regional routing, no anatomy penalty, and no routing-entropy penalty. Three evaluation-only conditions reused the signed full-model checkpoints: no temperature scaling, fixed thresholds of 0.5, and no hierarchical probability composition.

Two flat three-class controls were trained on the same partitions and model seeds. The conventional control uses the same pretrained ConvNeXt-Tiny backbone, global average pooling, layer normalization, dropout, and a linear three-class head. The architecture-matched flat control uses the mask-conditioned CALM-CXR regional-expert network with a single three-class output. Both flat models use validation macro-F1 for checkpoint selection and temperature scaling fitted only on the calibration partition. The flat models run for 20 epochs, whereas each hierarchical stage runs for 12 epochs, thus the comparison controls the data, backbone family, seeds, and evaluation protocol but not parameter count or total optimization steps.

### Metrics and statistical outputs

Per-seed outputs include accuracy, macro- and weighted F1, macro one-versus-rest AUROC, macro PR-AUC, 15-bin Expected Calibration Error, multiclass Brier score, negative log-likelihood, a confusion matrix, and class-wise precision, recall, and F1. Five hundred nonparametric bootstrap resamples provide test-set intervals for accuracy and macro-F1. Across seeds, the primary summary reports the mean and sample standard deviation. Definitions of these standard measures are not repeated, their exact implementation, including the 15 equal-width confidence bins used for ECE, is specified in the released source code and archived configuration.

Full hierarchical predictions are aligned with each flat baseline by the stored base index. For each seed, the package reports an exact two-sided McNemar test and paired-bootstrap differences in accuracy and macro-F1 with 95% percentile intervals. These paired tests assess the same 484 test images, they do not substitute for independent external validation.

### Execution and output structure

The runner saves one configuration and signature for each experiment, best and last checkpoints, training histories, calibration and test logit caches, metrics, prediction arrays, paired statistical results, compact revision tables and figures, per-seed manifests, an experiment registry, and an environment manifest. A successful rerun reuses an artifact only when the stored configuration, code, dataset, split, and checkpoint signatures match.

master_split/

experiments/<condition>/seed_<seed>/

baselines/<flat_model>/seed_<seed>/

statistics/

revision_tables/ revision_figures/

seed_manifests/ experiment_registry.json

environment_manifest.json review_revision_manifest.json

The final manifest confirms completion of seeds 42, 52, and 62 without a final failed seed. External-evaluation functions are implemented, but external_image_root was null in the completed configuration, therefore no independent external-validation result is claimed.

## Protocol validation

Protocol validation was performed as an internal method demonstration on the fixed filename-derived group-disjoint partition. All structural conditions, evaluation-only conditions, and flat controls used the same 484-image test partition and model seeds 42, 52, and 62. Results are reported as the mean (sample SD) across seeds. Because only three training seeds were available, component comparisons are descriptive, seed outputs were not treated as independent patient cohorts.

### Validation setting and output integrity

The primary manifest contained 4,840 images assigned to training (3,434), validation (435), calibration (487), and testing (484). No split_group_id occurred in more than one partition. These identifiers were derived from filenames rather than authenticated clinical records, the audit therefore establishes group disjointness, not verified patient-level separation.

The final manifest records successful completion of all requested seeds without a final failed seed. Thirty stored condition-seed result files were checked against their prediction arrays. Every file contained the same 484 base indices, filenames, and class labels, and recomputed accuracy and macro-F1 matched the archived summary values. The external-validation result table was empty.

### Full-protocol performance and error profile

The full protocol achieved accuracy 0.7920 (0.0248), macro-F1 0.7999 (0.0165), macro one-versus-rest AUROC 0.9063 (0.0088), macro PR-AUC 0.8616 (0.0068), ECE 0.0682 (0.0253), Brier score 0.3187 (0.0020), and negative log-likelihood 0.5772 (0.0117) as summarized in Table Table 1. The corresponding per-seed bootstrap intervals are retained in the supplementary metrics files, they were not pooled because each seed evaluated the same test images.

**Table 1:** Three-seed internal validation and structural controls. Values are mean (sample SD).

Condition	Accuracy	Macro-F1	AUROC	Macro PR-AUC	ECE
Full hierarchical protocol	0.7920 (0.0248)	0.7999 (0.0165)	0.9063 (0.0088)	0.8616 (0.0068)	0.0682 (0.0253)
No lung mask	0.7686 (0.0264)	0.7792 (0.0230)	0.8944 (0.0159)	0.8383 (0.0176)	0.0679 (0.0189)
Uniform regional routing	0.7803 (0.0160)	0.7895 (0.0112)	0.9023 (0.0061)	0.8484 (0.0020)	0.0401 (0.0075)
No anatomy penalty	0.7844 (0.0133)	0.7867 (0.0080)	0.9089 (0.0105)	0.8552 (0.0121)	0.0734 (0.0090)
No routing-entropy penalty	0.7975 (0.0169)	0.7951 (0.0196)	0.9088 (0.0002)	0.8549 (0.0037)	0.0370 (0.0213)
Flat ConvNeXt-Tiny	0.7955 (0.0135)	0.7982 (0.0128)	0.9124 (0.0026)	0.8638 (0.0032)	0.0423 (0.0052)
Flat CALM-CXR	0.7817 (0.0167)	0.7863 (0.0135)	0.8984 (0.0040)	0.8473 (0.0055)	0.0574 (0.0217)

Stage 1 remained substantially stronger than Stage 2. Across seeds, normal-abnormal screening achieved accuracy 0.9697 (0.0063) and macro-F1 0.9486 (0.0111), whereas bacteria-virus subtyping achieved accuracy 0.7764 (0.0314) and macro-F1 0.7514 (0.0252). Final class-wise F1 was 0.9156 (0.0183) for normal, 0.8131 (0.0272) for bacteria, and 0.6709 (0.0271) for virus. Of 302 final errors counted across the three seed-specific runs, 44 (14.6%) followed an incorrect Stage-1 decision and 258 (85.4%) occurred after correct abnormal routing. These repeated-test-set counts characterize the pathway of errors and are not independent case-frequency estimates.

### Structural component ablations

Removing lung-mask conditioning produced the largest mean decrease among the structural ablations: -0.0234 accuracy, -0.0206 macro-F1, -0.0119 AUROC, and -0.0233 macro PR-AUC relative to the full protocol. Uniform regional routing reduced accuracy by 0.0117, macro-F1 by 0.0103, and macro PR-AUC by 0.0132. Removing the anatomy penalty reduced macro-F1 by 0.0132 and macro PR-AUC by 0.0064. Removing the routing-entropy penalty reduced macro-F1 by 0.0048 and macro PR-AUC by 0.0066, although mean accuracy increased by 0.0055.

**Fig. 1:**
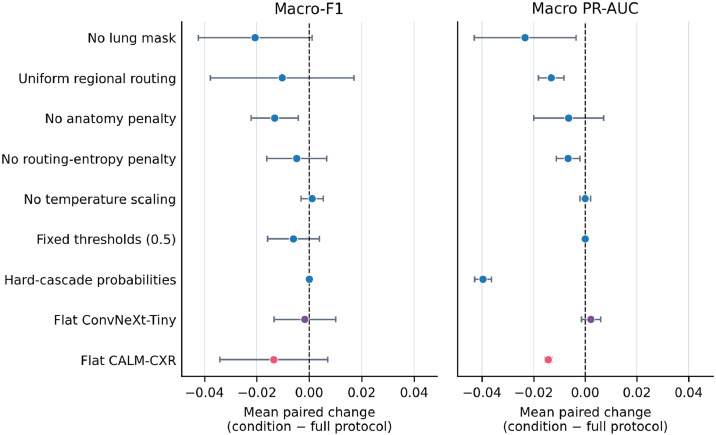
Mean paired changes relative to the full hierarchical protocol across seeds 42, 52, and 62. Whiskers are sample SD, negative values indicate a lower score than the full protocol. Classification labels are unchanged by the hard-cascade probability ablation, whereas its ranking scores change.

The effects were therefore not uniformly directional across metrics or seeds. The ablations support the largest and most consistent contribution from lung-mask conditioning and show smaller task-dependent roles for routing and the regularizers, they do not establish that every component improves every endpoint. Fig. 1 displays paired seed-wise changes rather than differences between unrelated summary means.

### Calibration, threshold selection, and probability composition

The fitted Stage-1 temperatures ranged from 0.6553 to 0.8383 and the Stage-2 temperatures from 1.7555 to 2.1391. Calibration-selected thresholds also varied across seeds (Stage 1, 0.415-0.850, Stage 2, 0.335-0.755), demonstrating that these quantities are model- and partition-dependent rather than transferable constants.

Without temperature scaling, accuracy and macro-F1 changed by only +0.0007 and +0.0011 on average, but Brier score increased by 0.0227 and negative log-likelihood by 0.1333. ECE decreased by 0.0084, so temperature scaling improved the two proper scoring rules but not every calibration summary. Replacing calibration-selected thresholds with 0.5 reduced accuracy by 0.0062 and macro-F1 by 0.0061 while leaving probability-based metrics unchanged. Replacing product composition with hard-cascade probabilities left the thresholded class labels unchanged by design, but reduced AUROC by 0.0107 and macro PR-AUC by 0.0395 and increased negative log-likelihood by 0.3172. Thus, hierarchical composition affected probability coherence and ranking rather than the stored decision labels. The effects of removing temperature scaling, fixing both thresholds at 0.5, and replacing soft hierarchical composition with hard-cascade probabilities are summarized in Table 2.

**Table 2:** Evaluation-only ablations. Values are mean (sample SD) across three seeds.

Condition	Accuracy	Macro-F1	AUROC	Macro PR-AUC	ECE	Brier	NLL
Full protocol	0.7920 (0.0248)	0.7999 (0.0165)	0.9063 (0.0088)	0.8616 (0.0068)	0.0682 (0.0253)	0.3187 (0.0020)	0.5772 (0.0117)
No temperature scaling	0.7927 (0.0217)	0.8010 (0.0128)	0.9065 (0.0080)	0.8616 (0.0084)	0.0597 (0.0094)	0.3414 (0.0039)	0.7105 (0.0128)
Fixed thresholds (0.5)	0.7858 (0.0102)	0.7938 (0.0103)	0.9063 (0.0088)	0.8616 (0.0068)	0.0682 (0.0253)	0.3187 (0.0020)	0.5772 (0.0117)
Hard-cascade probabilities	0.7920 (0.0248)	0.7999 (0.0165)	0.8957 (0.0056)	0.8220 (0.0061)	0.0570 (0.0273)	0.3210 (0.0029)	0.8944 (0.0908)

### Comparison with flat three-class controls

The conventional flat ConvNeXt-Tiny control achieved accuracy 0.7955 (0.0135), macro-F1 0.7982 (0.0128), AUROC 0.9124 (0.0026), and macro PR-AUC 0.8638 (0.0032). The architecture-matched flat CALM-CXR control achieved accuracy 0.7817 (0.0167), macro-F1 0.7863 (0.0135), AUROC 0.8984 (0.0040), and macro PR-AUC 0.8473 (0.0055). The hierarchical protocol was therefore similar to the conventional flat backbone and higher on the reported discrimination metrics than the architecture-matched flat control at the level of three-seed means as detailed in Table 3.

**Table 3:** Calibration parameters selected independently for each model seed. Calibration F1 is the macro-F1 used for threshold selection.

Seed	Stage-1 T	Stage-1 threshold	Stage-1 calibration F1	Stage-2 T	Stage-2 threshold	Stage-2 calibration F1
42	0.8383	0.415	0.9293	2.0090	0.335	0.7591
52	0.6553	0.850	0.9338	1.7555	0.565	0.7753
62	0.7188	0.685	0.9392	2.1391	0.755	0.7817

Prediction-level inference did not establish consistent superiority. For each seed and flat baseline, full-protocol and flat predictions were aligned on the same 484 test cases. Exact two-sided McNemar p-values were all greater than 0.05. Most 500-resample paired-bootstrap intervals for accuracy and macro-F1 included zero, the only positive accuracy interval was against flat CALM-CXR at seed 52, while its corresponding macro-F1 interval included zero and McNemar p was 0.0722 (results are reported in Table 4). The hierarchical organization is consequently supported as an auditable decomposition, not as a demonstrated universally superior classifier.

**Table 4:** Paired full-protocol minus flat-baseline differences on the 484 test images. Brackets are 95% percentile bootstrap intervals, p is the exact two-sided McNemar value.

Flat baseline	Seed	Accuracy difference [95% CI]	Macro-F1 difference [95% CI]	McNemar p
Flat ConvNeXt-Tiny	42	-0.0269 [-0.0640, 0.0073]	-0.0117 [-0.0439, 0.0212]	0.1766
Flat ConvNeXt-Tiny	52	0.0103 [-0.0145, 0.0351]	0.0100 [-0.0142, 0.0375]	0.5682
Flat ConvNeXt-Tiny	62	0.0062 [-0.0227, 0.0351]	0.0067 [-0.0252, 0.0355]	0.7838
Flat CALM-CXR	42	-0.0248 [-0.0579, 0.0041]	-0.0101 [-0.0387, 0.0192]	0.1619
Flat CALM-CXR	52	0.0310 [0.0010, 0.0640]	0.0251 [-0.0055, 0.0564]	0.0722
Flat CALM-CXR	62	0.0248 [-0.0041, 0.0496]	0.0257 [-0.0045, 0.0526]	0.1114

### External-validation status and evidential scope

No independent external dataset was configured: external_image_root was null, and the archived external-validation table contains no rows. The reported estimates therefore constitute internal validation on one filename-derived grouped split. Acquisition-site transportability, performance under authentic patient-level separation, and external three-class validity remain untested. The supplementary package retains the per-seed metrics, prediction arrays, split audit, paired statistical outputs, and source values for Fig. 1.

## Limitations

CALM-CXR is a research evaluation protocol, not an independently validated diagnostic system. The completed experiments used one ASCE-processed dataset and one fixed filename-derived group-disjoint split. Although the stored audit found no overlap among split_group_id values, those identifiers were inferred from filenames and were not authenticated against clinical patient records. Patient-level independence, acquisition-site transportability, and external three-class validity therefore remain untested. The 176 images without an acceptable filename-derived grouping basis were excluded from the primary analysis, which may also affect representativeness.

The bacteria, normal, and virus labels are dataset categories rather than microbiologically confirmed etymologies. Radiographic appearances of bacterial and viral pneumonia overlap, and the workflow lacks laboratory confirmation, clinical covariates, lesion-level annotations, and a radiologist-reader comparison. Consistent with this limitation, Stage 2 bacteria-virus subtyping was weaker than Stage 1 normal-abnormal screening, and virus had the lowest final class-wise F1. These results support error attribution within the dataset but not etiologic diagnosis from a radiograph.

Only three model seeds were evaluated, and all seeds used the same 484-image test partition. The across-seed means and sample standard deviations are consequently descriptive. The paired bootstrap intervals quantify prediction differences on this test set, while the McNemar tests assess paired label errors, neither addresses external generalizability. Component ablations were not subjected to multiple-comparison inference. In addition, the flat controls shared the data partitions, backbone family, seeds, and evaluation protocol but were not matched exactly for parameter count or total optimization steps. Non-significant paired comparisons should therefore not be interpreted as proof of equivalence.

The fitted temperatures and calibration-selected thresholds varied across seeds and should be re-estimated on a separate calibration partition whenever the protocol is transferred. Temperature scaling improved Brier score and negative log-likelihood on average but not ECE, illustrating that calibration conclusions depend on the metric. Finally, calibration and test logits were collected with one deterministic routing pass, so the stored test outputs do not evaluate Monte Carlo routing-uncertainty averaging. Multicentre external validation with authenticated patient identifiers, clinically verified labels, and reader comparison is required before any clinical-use claim.

## CRediT author statement

***Suresh Kumar Samarla:*** *Conceptualization, Methodology, Software, Data curation, Formal analysis, Investigation, Validation, Visualization, Writing – original draft, Writing – review & editing.*

**D. N. S. B. Kavitha**: Methodology, Data curation, Supervision, Writing – review & editing.

**Solleti Phanikumar:** Methodology, Investigation, Validation, Supervision, Writing – review & editing.

**Boddu L. V. Siva Rama Krishna:** Methodology, Investigation, Validation, Formal analysis, Writing – review & editing.

**D. S. S. Lakshmi Kumari P**.: Methodology, Investigation, Validation, Visualization, Writing – review & editing.

*All authors reviewed and approved the final version of the manuscript.*

Supplementary material *and/or* additional information

The broader ASCE-processed image dataset is openly available in Zenodo (version 1, 10.5281/zenodo.15542446). The present primary analysis used the audited 4,840-image filename-derived grouped subset specified in the accompanying metadata and split files.

The public implementation repository is available at https://github.com/samarlasureshkumar/CLAM-CXR. It documents the hierarchical workflow, calibration procedure, evaluation metrics, and expected dataset organization.

The reviewer-revision materials accompanying this manuscript contain the supplied execution script and notebook, together with a separate record of the two runtime corrections used in the completed runs, they also contain configuration and environment records, metadata and split audits, per-seed metrics and prediction arrays, paired statistical outputs, and revision tables and figures. Model checkpoints are not included in the compact archive because of their size.

## Declaration of competing interest

The authors declare that they have no known competing financial interests or personal relationships that could have appeared to influence the work reported in this paper.

## Data Availability

The data and supporting materials associated with this protocol are available in Zenodo at and https://zenodo.org/records/15542446 and https://github.com/samarlasureshkumar/CLAM-CXR.

## Appendix Supplementary materials

Image, application 1
