# Supplementary material for: CALM-CXR: a calibration-aware lung-masked workflow for hierarchical chest X-ray classification
Source: MethodsX. 2026 Jul 31;17:104081. doi: 10.1016/j.mex.2026.104081 (PMC13463977; doi:10.1016/j.mex.2026.104081)
Supplement: Supplementary file 1 [file mmc1.docx]

Supplementary Material

CALM-CXR: A Calibration-Aware Lung-Masked Workflow for Hierarchical Chest X-ray Classification

**Suresh Kumar Samarla et al.**

*Purpose: workflow clarification, cohort provenance, reviewer-requested ablations, matched flat controls, and reproducibility details.*

# How to read this supplement

This supplement expands the operational detail behind the revised CALM-CXR protocol. Figure S1 explains the end-to-end data flow; Tables S1–S4 define the cohort, components, experimental controls, and implementation settings; Figures S2–S3 and Tables S5–S10 provide the reviewer-requested component, calibration, threshold, probability-composition, and flat-baseline comparisons. All primary quantitative conclusions refer to the revised 4,840-image cohort and its fixed 484-image test partition.

**Interpretive boundary.** The hierarchy was selected for stage-wise auditability, not because it was assumed to be intrinsically superior. Results from the originally submitted analysis are superseded because the historical and revised evaluations used different cohort definitions and test partitions.

# S1. End-to-end workflow

CALM-CXR separates representation learning, model selection, probability calibration, decision-threshold selection, and final test evaluation. The same mask-conditioned regional architecture is trained independently for Stage 1 and Stage 2. Validation macro-F1 selects checkpoints. The calibration partition alone estimates stage-specific temperatures and thresholds. The untouched test partition is used once for the reported metrics.

**Supplementary Figure S1.** Operational CALM-CXR workflow.

A lung mask conditions the regional representation. Stage 1 estimates normal versus abnormal; Stage 2 estimates bacteria versus virus conditional on abnormality. Calibration-only parameters are fitted separately for the two stages, after which three coherent class probabilities are composed.

# S2. Cohort construction and partition provenance

The revised primary cohort contains 4,840 images. A split_group_id was derived from documented filename patterns for 2,481 groups, and complete groups were allocated to exactly one partition. Because those identifiers were not authenticated against clinical records, the split is described as filename-derived group-disjoint rather than verified patient-level.

**Supplementary Table S1.** Primary cohort and fixed master split.

| **Partition** | **Images** | **Filename-derived groups** | **Bacteria** | **Normal** | **Virus** |
| --- | --- | --- | --- | --- | --- |
| Training | 3434 | 1736 | 1842 | 599 | 993 |
| Validation | 435 | 248 | 219 | 87 | 129 |
| Calibration | 487 | 248 | 255 | 85 | 147 |
| Testing | 484 | 249 | 271 | 86 | 127 |
| Total | 4840 | 2481 | 2587 | 857 | 1396 |

***Note.*** *No split_group_id occurred in more than one partition. Group identifiers were filename-derived and were not authenticated clinical patient identifiers.*

An additional 176 publicly sourced chest X-rays used in an earlier study were kept outside the 4,840-image primary cohort. They were not included in the revised training, validation, calibration, or test partitions and were not evaluated as an independent external cohort using the revised checkpoints. Accordingly, the present study makes no external-validation performance claim.

# S3. What each protocol component contributes

**Supplementary Table S2.** Protocol components, computational roles, and corresponding controls.

| **Component** | **Operational role** | **Control** |
| --- | --- | --- |
| Lung-mask conditioning | Conditions feature extraction and lung-grid occupancy; not merely image cropping | full_protocol versus no_lung_mask |
| Regional expert routing | Learns spatially varying expert allocation over lung-region tokens | full_protocol versus no_regional_routing |
| Anatomy penalty | Penalizes confident routing outside lung occupancy | full_protocol versus no_anatomy_penalty |
| Routing entropy penalty | Controls diffuse expert allocation inside lung regions | full_protocol versus no_entropy_penalty |
| Stage-wise temperature scaling | Calibrates normal-abnormal and bacteria-virus probabilities independently | full_protocol versus no_temperature_scaling |
| Stage-wise threshold optimization | Fits decision thresholds only on the calibration partition | full_protocol versus fixed_thresholds_0.5 |
| Hierarchical probability composition | Propagates Stage-1 abnormal probability into final subtype probabilities | full_protocol versus no_hierarchical_probability_composition |
| Hierarchical organization | Separates screening and subtype errors and supports stage-wise error attribution | full_protocol versus flat_calm_cxr and flat_backbone |

***Note.*** *Structural controls were retrained independently for each seed. Evaluation-only controls reused the corresponding signed full-model checkpoints.*

# S4. Experimental conditions and fairness of comparisons

**Supplementary Table S3.** Reviewer-oriented experimental conditions.

| **Condition** | **Execution** | **Change relative to full protocol** |
| --- | --- | --- |
| Full protocol | Retrained | Mask conditioning, learned routing, anatomy and entropy penalties, stage-wise calibration, optimized thresholds, product composition |
| No lung mask | Retrained | Mask conditioning removed |
| Uniform regional routing | Retrained | Learned routing replaced by uniform expert allocation |
| No anatomy penalty | Retrained | Anatomy regularization coefficient set to zero |
| No routing-entropy penalty | Retrained | Routing-entropy coefficient set to zero |
| No temperature scaling | Evaluation only | Uncalibrated probabilities from full-model checkpoints |
| Fixed thresholds 0.5 | Evaluation only | Calibration-derived decision thresholds replaced with 0.5 |
| No hierarchical composition | Evaluation only | Stage probabilities not combined by the product rule |
| Flat ConvNeXt-Tiny | Retrained flat baseline | Same pretrained backbone family with global average pooling and a linear three-class head |
| Flat CALM-CXR architecture | Retrained flat baseline | Mask-conditioned regional-expert network with one three-class output |

***Note.*** *All conditions used the same master partitions and model seeds 42, 52, and 62. Flat models trained for 20 epochs; each hierarchical stage trained for 12 epochs. Parameter count and total optimization steps were therefore not matched.*

**Supplementary Table S4.** Key implementation and evaluation settings.

| **Category** | **Setting** |
| --- | --- |
| Input / backbone | 224 × 224; pretrained ConvNeXt-Tiny |
| Regional model | 7 × 7 grid; 3 experts; expert dimension 256; router hidden dimension 256 |
| Regularization | Dropout 0.20; label smoothing 0.05; anatomy λ=0.05; routing entropy λ=0.01; conflict λ=0.01 |
| Optimization | Batch 16; backbone LR 1×10⁻⁵; head LR 2×10⁻⁴; weight decay 1×10⁻⁴ |
| Training schedule | Backbone frozen for 3 epochs; Stage 1 and Stage 2: 12 epochs; flat controls: 20 epochs |
| Sampling / precision | Weighted sampler; automatic mixed precision |
| Uncertainty / inference | 6 Monte Carlo samples; no horizontal-flip test-time augmentation |
| Selection / calibration | Validation macro-F1 checkpoint selection; temperatures and thresholds fitted only on calibration partition |
| Uncertainty intervals | 500 bootstrap resamples for within-run accuracy and macro-F1 intervals |

***Note.*** *These settings were held fixed across the reviewer-oriented experiments except where a component was deliberately ablated.*

# S5. Full-protocol performance across seeds

**Supplementary Table S5.** Full-protocol discrimination and calibration metrics.

| **Seed** | **Accuracy** | **Macro-F1** | **Weighted-F1** | **AUROC** | **PR-AUC** | **ECE** | **Brier** | **NLL** |
| --- | --- | --- | --- | --- | --- | --- | --- | --- |
| 42 | 0.7665 | 0.7857 | 0.7728 | 0.9063 | 0.8621 | 0.0582 | 0.3196 | 0.5736 |
| 52 | 0.7934 | 0.7959 | 0.7940 | 0.8975 | 0.8545 | 0.0969 | 0.3201 | 0.5903 |
| 62 | 0.8161 | 0.8180 | 0.8152 | 0.9152 | 0.8681 | 0.0493 | 0.3165 | 0.5677 |
| Mean ± SD | 0.7920 ± 0.0248 | 0.7999 ± 0.0165 | 0.7940 ± 0.0212 | 0.9063 ± 0.0088 | 0.8616 ± 0.0068 | 0.0682 ± 0.0253 | 0.3187 ± 0.0020 | 0.5772 ± 0.0117 |

***Note.*** *AUROC is macro one-versus-rest; PR-AUC is macro averaged. SD is the sample standard deviation across three model seeds. Lower ECE, Brier score, and NLL indicate better probability calibration.*

# S6. Structural component ablations

**Supplementary Table S6.** Mean structural-ablation results across seeds.

| **Condition** | **Accuracy** | **Δ Acc.** | **Macro-F1** | **Δ F1** | **AUROC** | **PR-AUC** |
| --- | --- | --- | --- | --- | --- | --- |
| Full protocol | 0.7920 ± 0.0248 | 0.0000 | 0.7999 ± 0.0165 | 0.0000 | 0.9063 ± 0.0088 | 0.8616 ± 0.0068 |
| No lung mask | 0.7686 ± 0.0264 | 0.0234 | 0.7792 ± 0.0230 | 0.0206 | 0.8944 ± 0.0159 | 0.8383 ± 0.0176 |
| Uniform regional routing | 0.7803 ± 0.0160 | 0.0117 | 0.7895 ± 0.0112 | 0.0103 | 0.9023 ± 0.0061 | 0.8484 ± 0.0020 |
| No anatomy penalty | 0.7844 ± 0.0133 | 0.0076 | 0.7867 ± 0.0080 | 0.0132 | 0.9089 ± 0.0105 | 0.8552 ± 0.0121 |
| No routing-entropy penalty | 0.7975 ± 0.0169 | -0.0055 | 0.7951 ± 0.0196 | 0.0048 | 0.9088 ± 0.0002 | 0.8549 ± 0.0037 |

***Note.*** *Δ values are full protocol minus ablation; positive values indicate a mean decrease after component removal. With only three seeds, these differences should be interpreted descriptively.*

**Supplementary Figure S2.** Mean changes after structural component removal.

Removing lung-mask conditioning produced the largest mean decrease in accuracy (0.0234) and macro-F1 (0.0206). Negative bars indicate that the ablated condition had a slightly higher mean for that metric; these small three-seed differences are not evidence of superiority.

# S7. Evaluation-only controls

**Supplementary Table S7.** Evaluation-only ablations using the same full-model checkpoints.

| **Condition** | **Accuracy** | **Macro-F1** | **AUROC** | **PR-AUC** | **ECE** | **Brier** | **NLL** |
| --- | --- | --- | --- | --- | --- | --- | --- |
| Full protocol | 0.7920 ± 0.0248 | 0.7999 ± 0.0165 | 0.9063 ± 0.0088 | 0.8616 ± 0.0068 | 0.0682 ± 0.0253 | 0.3187 ± 0.0020 | 0.5772 ± 0.0117 |
| No temperature scaling | 0.7927 ± 0.0217 | 0.8010 ± 0.0128 | 0.9065 ± 0.0080 | 0.8616 ± 0.0084 | 0.0597 ± 0.0094 | 0.3414 ± 0.0039 | 0.7105 ± 0.0128 |
| Fixed thresholds of 0.5 | 0.7858 ± 0.0102 | 0.7938 ± 0.0103 | 0.9063 ± 0.0088 | 0.8616 ± 0.0068 | 0.0682 ± 0.0253 | 0.3187 ± 0.0020 | 0.5772 ± 0.0117 |
| No hierarchical composition | 0.7920 ± 0.0248 | 0.7999 ± 0.0165 | 0.8957 ± 0.0056 | 0.8220 ± 0.0061 | 0.0570 ± 0.0273 | 0.3210 ± 0.0029 | 0.8944 ± 0.0908 |

***Note.*** *Temperature scaling mainly affected proper scoring rules: removing it increased mean Brier score from 0.3187 to 0.3414 and mean NLL from 0.5772 to 0.7105. Removing hierarchical composition left threshold-based labels unchanged in this implementation but degraded AUROC, PR-AUC, and NLL because the reported probability vectors changed.*

# S8. Matched flat-classifier baselines

**Supplementary Table S8.** Hierarchical and flat three-class results across seeds.

| **Model** | **Accuracy** | **Macro-F1** | **AUROC** | **PR-AUC** | **ECE** | **Brier** | **NLL** |
| --- | --- | --- | --- | --- | --- | --- | --- |
| Hierarchical CALM-CXR | 0.7920 ± 0.0248 | 0.7999 ± 0.0165 | 0.9063 ± 0.0088 | 0.8616 ± 0.0068 | 0.0682 ± 0.0253 | 0.3187 ± 0.0020 | 0.5772 ± 0.0117 |
| Flat ConvNeXt-Tiny | 0.7955 ± 0.0135 | 0.7982 ± 0.0128 | 0.9124 ± 0.0026 | 0.8638 ± 0.0032 | 0.0423 ± 0.0052 | 0.2957 ± 0.0077 | 0.5343 ± 0.0121 |
| Flat CALM-CXR architecture | 0.7817 ± 0.0167 | 0.7863 ± 0.0135 | 0.8984 ± 0.0040 | 0.8473 ± 0.0055 | 0.0574 ± 0.0217 | 0.3298 ± 0.0247 | 0.5868 ± 0.0264 |

***Note.*** *The flat ConvNeXt-Tiny control slightly exceeded the hierarchical mean on several endpoint metrics, whereas the hierarchy exposes stage-specific decisions and error attribution. The comparison matches data partitions, backbone family, seeds, and evaluation procedure, but not parameter count or total optimization steps.*

**Supplementary Figure S3.** Comparison with matched flat classifiers.

Bars show the mean across seeds 42, 52, and 62. The comparison does not establish consistent overall predictive superiority of the hierarchical organization; its principal advantage in this protocol is auditability.

**Supplementary Table S9.** Paired prediction-level comparisons: hierarchy minus flat control.

| **Seed** | **Flat control** | **H✓/F✗** | **H✗/F✓** | **McNemar p** | **Δ Acc.** | **95% CI** | **Δ F1** | **95% CI** |
| --- | --- | --- | --- | --- | --- | --- | --- | --- |
| 42 | Flat ConvNeXt-Tiny | 33 | 46 | 0.177 | -0.0269 | [-0.0640, 0.0073] | -0.0117 | [-0.0439, 0.0212] |
| 42 | Flat CALM-CXR architecture | 25 | 37 | 0.162 | -0.0248 | [-0.0579, 0.0041] | -0.0101 | [-0.0387, 0.0192] |
| 52 | Flat ConvNeXt-Tiny | 27 | 22 | 0.568 | 0.0103 | [-0.0145, 0.0351] | 0.0100 | [-0.0142, 0.0375] |
| 52 | Flat CALM-CXR architecture | 38 | 23 | 0.072 | 0.0310 | [0.0010, 0.0640] | 0.0251 | [-0.0055, 0.0564] |
| 62 | Flat ConvNeXt-Tiny | 28 | 25 | 0.784 | 0.0062 | [-0.0227, 0.0351] | 0.0067 | [-0.0252, 0.0355] |
| 62 | Flat CALM-CXR architecture | 30 | 18 | 0.111 | 0.0248 | [-0.0041, 0.0496] | 0.0257 | [-0.0045, 0.0526] |

***Note.*** *H denotes hierarchical CALM-CXR and F denotes the named flat control. CIs are paired bootstrap intervals over the 484 shared test cases. No correction for multiple comparisons was applied; results are supporting analyses rather than confirmatory hypothesis tests.*

# S9. Calibration and decision parameters

**Supplementary Table S10.** Stage-wise calibration parameters and final test calibration metrics.

| **Seed** | **T₁** | **T₂** | **τ₁** | **τ₂** | **Cal. F1₁** | **Cal. F1₂** | **ECE** | **Brier** | **NLL** |
| --- | --- | --- | --- | --- | --- | --- | --- | --- | --- |
| 42 | 0.8383 | 2.0090 | 0.415 | 0.335 | 0.9293 | 0.7591 | 0.0582 | 0.3196 | 0.5736 |
| 52 | 0.6553 | 1.7555 | 0.850 | 0.565 | 0.9338 | 0.7753 | 0.0969 | 0.3201 | 0.5903 |
| 62 | 0.7188 | 2.1391 | 0.685 | 0.755 | 0.9392 | 0.7817 | 0.0493 | 0.3165 | 0.5677 |

***Note.*** *T denotes temperature and τ denotes the calibration-selected decision threshold. These parameters were estimated using the calibration partition only. Calibration macro-F1 values are partition-specific selection summaries, not test performance.*

# S10. Reproducibility and provenance notes

All reviewer-oriented experiments used the same fixed master split and model seeds 42, 52, and 62. The reproducibility package provides per-seed configurations, signed checkpoints, metrics files, saved predictions, the split manifest, aggregate result tables, and paired-comparison outputs. The 484-image test partition was reserved for final evaluation.

**Split terminology.** The grouping variable was derived from documented filename patterns and was not authenticated against clinical patient records. It therefore supports a filename-derived group-disjoint claim, not a verified patient-level claim. Machine-readable fields that use patient-oriented labels should be interpreted as referring to filename-derived groups.

**External-validation boundary.** The 176 publicly sourced images used in an earlier study were excluded from the revised primary cohort and were not evaluated with the revised checkpoints as an independent external cohort. No external-validation performance is claimed.

**Historical result.** The originally submitted single-run estimate is superseded because it used a different cohort definition and test partition. It is not pooled or statistically compared with the revised three-seed results.

# Abbreviations

AUROC, area under the receiver-operating-characteristic curve; CXR, chest X-ray; ECE, expected calibration error; F1, harmonic mean of precision and recall; NLL, negative log-likelihood; PR-AUC, area under the precision–recall curve; SD, standard deviation.
